# Why People Do, or Do Not, Immediately Contact Emergency Medical Services following the Onset of Acute Stroke: Qualitative Interview Study

**DOI:** 10.1371/journal.pone.0046124

**Published:** 2012-10-04

**Authors:** Joan E. Mackintosh, Madeleine J. Murtagh, Helen Rodgers, Richard G. Thomson, Gary A. Ford, Martin White

**Affiliations:** 1 Institute of Health and Society, Faculty of Medical Sciences, Newcastle University, Newcastle upon Tyne, United Kingdom; 2 Department of Health Sciences, College of Medicine, Biological Sciences and Psychology, Leicester University, Leicester, United Kingdom; 3 Institute for Ageing and Health, Faculty of Medical Sciences, Newcastle University, Newcastle upon Tyne, United Kingdom; 4 Fuse, UKCRC Centre for Translational Research in Public Health, Newcastle University, Newcastle upon Tyne, United Kingdom; Cardiff University, United Kingdom

## Abstract

**Objectives:**

To identify the reasons why individuals contact, or delay contacting, emergency medical services in response to stroke symptoms.

**Design:**

Qualitative interview study with a purposive sample of stroke patients and witnesses, selected according to method of accessing medical care and the time taken to do so. Data were analysed using the Framework approach.

**Setting:**

Area covered by three acute stroke units in the north east of England.

**Participants:**

Nineteen stroke patients and 26 witnesses who had called for help following the onset of stroke symptoms.

**Results:**

Factors influencing who called emergency medical services and when they called included stroke severity, how people made sense of symptoms and their level of motivation to seek help. Fear of the consequences of stroke, including future dependence or disruption to family life, previous negative experience of hospitals, or involving a friend or relations in the decision to access medical services, all resulted in delayed admission. Lack of knowledge of stroke symptoms was also an important determinant. Perceptions of the remit of medical services were a major cause of delays in admission, with many people believing the most appropriate action was to telephone their GP. Variations in the response of primary care teams to acute stroke symptoms were also evident.

**Conclusions:**

The factors influencing help-seeking decisions are complex. There remains a need to improve recognition by patients, witnesses and health care staff of the need to treat stroke as a medical emergency by calling emergency medical services, as well as increasing knowledge of symptoms of stroke among patients and potential witnesses. Fear, denial and reticence to impose on others hinders the process of seeking help and will need addressing specifically with appropriate interventions. Variability in how primary care services respond to stroke needs further investigation to inform interventions to promote best practice.

**Trial Registration:**

UK Clinical Research Network UKCRN 6590

## Introduction

Rapid admission to hospital following stroke is vital in ensuring patients have timely access to treatments such as thrombolysis. Thrombolysis with intravenous tissue Plasminogen Activator (tPA), when given to carefully selected patients within 4.5 hours of the onset of symptoms of acute ischaemic stroke, reduces the risk of dependency. [1], [2] It is estimated that 15–20% of acute ischaemic stroke patients should be eligible for thrombolysis, [3] but currently only 3.8% of patients in the UK receive this treatment. [4] One of the main reasons for the low rates of thrombolytic treatment is the lack of an urgent response to stroke symptoms by many patients and witnesses. [5]–[9] Early recognition and rapid response to the symptoms of stroke by patients and bystanders are important pre-requisites for improving outcome following stroke. [10]

A key component of the English National Stroke Strategy is to ensure that patients with acute stroke are treated as a medical emergency. [10] The strategy seeks to ensure that “members of the public and health and care staff are able to recognise and identify the main symptoms of stroke and know it needs to be treated as an emergency”. [10] In response to this, the Department of Health introduced the ‘Stroke - Act FAST’ awareness raising campaign in February 2009. [11]

In a recent systematic review, [12] we found limited evidence suggesting a good level of knowledge of the two commonest stroke symptoms (unilateral weakness and speech disturbance), and of the need for an urgent response among the public and at risk patients. However, whilst members of the public said that they would call an ambulance in the event of a stroke, in practice both patients and witnesses often initially contact a general practitioner, which significantly increases the time from symptom onset to admission. [12], [13] In addition, a substantial proportion of patients and witnesses wait to see if symptoms resolve before seeking help. Thus, although people report that they consider stroke to be a medical emergency, observed behaviour suggests this knowledge does not always result in an appropriate response. [12]

Presently it is unknown what factors influence how and why help is sought following the onset of stroke symptoms. The aim of the study was therefore to explore the reasons why people with stroke, and witnesses to their stroke, immediately contact, or delay contacting, emergency medical services in response to the onset of stroke symptoms.

## Methods

The protocol for this trial and supporting COREQ checklist are available as supporting information; see Checklist S1 and Protocol S1.

We investigated how, why and when emergency medical services are accessed by people with stroke and ‘witnesses’ (e.g. family members/friends/carers/bystanders who were present at the time of stroke or who found the patient with stroke and who made initial contact with medical services following the onset of acute stroke).

Audio-recorded, semi-structured interviews (lasting 12–40 minutes) were undertaken within 14 days of acute stroke with patients and witnesses to the stroke using topic guides developed utilising the results of our systematic review. [12] Thirty six interviews (22 witnesses, 14 patients) took place in participants' own homes, seven (two witnesses, five patients) on stroke units, one witness interview was conducted at her place of work and one witness interview was by telephone to her place of work. Interviews covered the context in which the stroke occurred, the symptoms experienced or witnessed, behavioural responses to the stroke by the patient and witnesses, prior knowledge of stroke symptoms and available treatments, views about contacting medical services, and awareness of and perspectives on the Act FAST campaign. [12], [13]

Patients and witnesses were purposively selected from three stroke units in north east England to include those who had accessed medical services in the following ways: called emergency medical services or attended an accident and emergency department within one hour, or after one hour, of onset of symptoms; or called primary care services (GP, out of hours service or NHS Direct telephone line) within one hour, or after one hour, of the onset of symptoms. Potential participants were approached by stroke research nurses and the names of interested individuals were passed to JM. It was not possible to ascertain how many people declined to participate.

Field notes were made by the researcher after the interviews and sampling continued until no new themes emerged from the data. JM and MJM undertook thematic analysis following the Framework method, [14] with constant comparison [15] and deviant case analysis [16] to enhance internal validity. Resulting typologies were derived, and descriptive and explanatory categories developed. JM (BSc) is an experienced health services researcher with 11 years qualitative research experience, and MJM (PhD) is a social scientist with 19 years qualitative research experience.

## Results

Nineteen patients and 26 witnesses were interviewed (Table 1). The patients were aged 41 to 86 years. In ten of the witness interviews, the stroke patient was present and made a contribution to the discussion. Forty of the 45 strokes occurred in patients' own homes. In seven cases there had been at least one previous stroke and 15 of the patients had some pre-stroke disability. One patient lived in sheltered housing and one in a care home where they experienced their strokes: in each of these cases a formal carer noticed the initial stroke symptoms and sought medical help. Two of the strokes occurred when patients were at work, one happened in a supermarket, one while the patient was out walking and one on a bus.

**Table 1:** Initial action taken by participants at onset of stroke symptoms.

Action taken at the onset of stroke symptoms	By patients	By witnesses
	Number (%)	Number (%)
Called emergency medical services within 1 hour	5 (26)	13 (50)
Called emergency medical services after 1 hour	3 (16)	7 (27)
Called GP within 1 hour	0	1 (4)
Called GP after 1 hour	9 (47)	3 (11)
Travelled independently to A&E after 1 hour	2 (11)	2 (8)

The process that leads to a decision about why people do, or do not, immediately contact emergency medical services after the onset of stroke involves a complex interaction of different factors. We identified the following five major themes that were found to be important in affecting the decisions of patients and witnesses.

### Interpreting the signs and symptoms of stroke

How people made sense of what was happening to them was an important factor in determining their actions. For some patients, particularly those who had experience of stroke in family members, there was a strong desire to get help quickly and they sought help as soon as they realised what was happening:

*“I was just starting my lunch when the fork dropped out of my hand and my friend that does a bit of housework for me she said “what's the matter?” I said “you might not believe it but I'm having a stroke, will you ring for the paramedics?” “I've got pins and needles and my hand's dropped, my arm's dropped and I can't feel anything”. So she rang straight away… I says “Tell her it's for an elderly gentleman, 75“ and how did I know it was a stroke the lady had said. I said “Because my wife used to have strokes, little mini strokes and I nursed her for 9 years and I knew how it went”.* (**C05P, male patient**)

For others, attempts to match their symptoms to what they knew about illness, and stroke in particular, often resulted in patients misinterpreting their symptoms:

*I thought it would be a sharp headache or something and then losing one part, the side of your body or something you know, your arm or leg you couldn't use it or something, that's what I expected a stroke to be* (**C06P, female patient**)

*I just thought I'd trapped a nerve… ‘cause I couldn't move my shoulder that was it…. that's why I didn't (go) to the hospital straight away* (**A03P, Male Patient**)

For many patients, the symptoms they initially experienced were considered mild and not readily recognised as stroke. Patients reported feeling “not quite right” or “fuzzy”. They did not think to call emergency services because they did not feel their symptoms were severe enough. There was an expectation that stroke would involve a clear and dramatic event.

*When I had my first one I was amazed, ‘cause I didn't realise it could happen just like that with your speech. When I got to hospital and they told me, I thought well I didn't feel anything and you know I knew what I wanted to say* (**A02,** f**emale patient**)

### Responses to symptoms of stroke

Several of the patients who had suffered previous strokes suspected that this was happening but did not call 999.

*I …What did you think was happening to you at the time?*

*P I knew*

*I You knew it was another stroke?*

*P Yes. I suspected.*

*I So [were] the symptoms similar to what you had the last time?*

*P [Yes]*

*I So what were you going to do about the symptoms you were having?*

*P I was hoping they would go away*

*I Right, you were just hoping they would go away of their own accord?*

*P Yeah* **(C03, male patient)**

Others, with no previous experience of stroke, suspected a stroke but decided to wait to see if the symptoms resolved spontaneously.

*When [my husband] came in I told him I thought I had had a stroke and he said ‘shall I take you to the hospital?’ and I said ‘well I don't feel too bad so I'll not go today’ but of course by Monday nothing had changed I wasn't any better and I thought well obviously it's sensible to go now to check if I am not getting any better at least we might find out what it was.* (**A07, female patient**)

A number of patients were adamant that they did not want to seek emergency medical help, with reasons including a longstanding fear of hospitals and not wanting to ‘bother’ medical services.

*I no ok before this happened what did you know about the signs and symptoms of stroke*

*P the adverts ha ha*

*I right so that's what you knew*

*P I still paid no attention to it cause of the fear of hospitals*

*P I've done all the health and safety thing, first aid things like that I know all about them through different courses and things like that*

*I you'd done that all for work ok but that didn't that still didn't affect your actions…?*

*P fear of hospitals is stronger* **(A03P, male patient)**

*P But it's a difficult one though isn't it? Because…I wouldn't want to dial 999, I wouldn't want anybody to dial 999 for me when I think there's nothing the matter, do you know what…? It's a difficult one really.* **(A05P, female patient)**

There was concern about taking up the time and resources of emergency services for symptoms that did not appear to be urgent and which might be better used for other ‘more deserving’ cases. Some patients seemed to have tried to ignore their stroke symptoms, reportedly because of anxiety about the possible consequences and the potential impact on their quality of life.

### Deflection and delay

Patients often contacted a relation or friend in the first instance. For some patients, even though they had suffered a previous stroke or were aware that they were having a stroke, their understanding of the urgency with which a response was needed was limited and it was more important to them to seek affirmation from others.

*I was crawling about and… of course I couldn't use my arm properly. I seemed to be there for hours and I knew I should do something, see I couldn't move about freely and err I didn't know just how long I was there because it was getting on to about 7 o'clock in the morning. I thought I better ring somebody so I rang my nephew who lives 10 minutes walk away and he came over straight away and he realised instantly I should have already got an ambulance.* **(C02P, male patient)**

Patients were keen to have a friend or relative not only to provide comfort and reassurance, but also to take responsibility for engaging with emergency medical services. The extra time taken and consequent implications for treatment were apparently less important than having someone they knew and trusted to take control.

*I When your friend you say it was that called the ambulance….how did you feel about that?*

*P I was very pleased that she took responsibility. I mean she's slightly younger than I am, but you could tell that she took responsibility for me*

*I And that was important to you?*

*P It was important and she stayed, she followed the ambulance and took me right through the A&E and then took me to the bedroom so what time she got home that morning I don't know but she's very nice* **(C01P, female patient)**

For witnesses, a transfer of responsibility occurred between themselves and health professionals. Seeking the reassurance of medical expertise in dealing with stroke transferred the responsibility for the consequences of the stroke and action or inaction at its onset, thus leading to rapid contact with emergency medical services.

*I think that the most important thing was em, I just needed help medical help for dad because when something like that happens you're out of your depth you don't know how to cope… you need somebody professional person you know.* **(A08W, female witness)**

The possibility that the patient could die as a result of the stroke played on the minds of both witnesses and patients. Patients reported that the degree of concern was linked with the severity of the stroke. For witnesses, however, the link between stroke and death was very strong and more often prompted them to contact emergency medical services regardless of severity.

*I um what were your main concerns or worries at the point when [patient] had the stroke*

*W I didn't want him to die*

*I right*

*W I didn't want him to leave me* **(A06W, female witness)**

The perception that the stroke, or potential future strokes, might result in permanent paralysis that would lead to the patient becoming disabled or dependent was a major concern for both witnesses and patients, but was voiced more often by witnesses.

*She was frightened she was going to have another one and she would lose… that she'd be like… well this sounds horrible but, you know, like couldn't do anything… paralysed and couldn't do anything at all* (**A06P, female patient**)

The desire for a long and healthy life, and to avoid death and disability as a result of a stroke, was given as an explanation by some patients as to why they immediately contacted emergency services at the onset of a stroke.

*I Okay, so you knew it was a stroke and you decided to…get to the hospital. What were the important factors to you in making that decision? What were you thinking about?*

*P Myself, you know, I want to be here a long time. I want to be shot by a jealous lover when I'm about 95 and survive.*

*I [Laughter], yes I bet.*

*P No, I like life and I look after myself you know, I mean I'm the one when I cross the road I look more than once each way.* **(C05P, male patient)**

Paradoxically, the fear of potential dependence was also cited as a reason why some people delayed calling for help, as their concern about possible consequences led them to deny their symptoms – at least in the short term.

### Prior knowledge and awareness of the Act FAST campaign

For many participants, knowledge of stroke symptoms had been gathered over years of observation or familial experience. The symptoms that participants in this study most associated with stroke were one-sided weakness, twisting or drooping of the mouth and confusion.

*Erm…well a couple of people over the years like older people that maybes you had worked with and their men had a stroke or she had a stroke. And seeing them with this twisted mouth. And sometimes with a stick and funny with their walking.* **(C05W, female witness)**

The impact of the UK Department of Health's Act FAST awareness raising campaign^11^ varied considerably amongst the participants in the study who were aware of it, but a number reported that it had increased their recognition of stroke symptoms. The ordinariness of the situations in the advertising campaign (e.g. a man at a sporting event) was something that was identified by a number of participants as helpful in making them realise that stroke could happen to anyone at any time and was not just a problem for the very elderly.

*Had it not been for the adverts, erm then I wouldn't have realised one of the major things that help you recognise the first signs of stroke is that the fact that it can happen to you and everyone there on those adverts, you know, you can see they are ordinary, normal people and they wouldn't expect it to happen to them or you wouldn't even expect it to happen to them, someone in a football match or something like that.* **(B02P, male patient)**

However, the experience of stroke did not always match up to participants' expectations. Not all of the participants experienced or witnessed all (or indeed any) of the symptoms shown in the campaign, and therefore did not recognise that a stroke was occurring. For a number of participants, who had not experienced or witnessed the ‘classic symptoms’ of stroke as alluded to in the campaign, the fact that stroke could present in other ways was surprising.

*I And before this happened what did you know about the signs and symptoms of stroke?*

*P Just really what you see on that advert.*

*I Right.*

*P Yeah, as I say, I think you just think that it's going to be no speech and you're really bad, that's the impression that I got.* **(A05P, female patient)**

Apart from the awareness raising campaign, other types of television programme, including popular culture (e.g. soap operas) were reported by participants as a source of knowledge.

*I guess I've probably read bits in papers and on TV erm as well like the actor in Eastenders had one* **(A10W, female witness)**

### The roles and responses of medical services

Peoples' beliefs and attitudes about which medical services to contact and under which circumstances were important determinants of actions. Many considered that emergency services were for major trauma or collapse only, and the patient's ability to continue to function, albeit at a reduced level, along with lack of pain, did not fit the criteria that they considered warranted an emergency response. For many, the most appropriate action was to contact their GP. This was particularly true for those with chronic illness for whom their relationship with the GP was one of trust which had been built up over a number of years.

*I Yeah, em, so obviously you decided to eh, ring your GP, why did you decide to do that?*

*P Just to see him to see what he thought* **(B01P, female patient)**

*I Okay, when you did decide to go to see your GP, why did you decide to do that?*

*P To find out what it was. I thought I had to go there before going to a hospital, I didn't realise that I could just go straight to the hospital. It didn't occur to me to do that* **(A07P, female patient)**

There was wide variation in the way participants reported that primary care services responded to a patient presenting with stroke symptoms. If staff were told that there was a suspected stroke, they reportedly advised urgent transfer to hospital. Delays appear to have occurred when the symptoms were milder or less obvious. There were times when GPs referred patients for hospital care but did not stress the urgency of the situation or make appropriate arrangements to get them to hospital immediately. One patient's symptoms were worsening as she went home on the bus from her GP's surgery to await the arrival of a GP-booked ambulance at home.

*I Who had asked for the ambulance to come?*

*P Well, it must have been the doctor*

*I So he'd let you go home on the bus and then rang an ambulance?*

*P Mind, I had a job to, when I got off, it must have been coming on more severe because I had a bit of a job to get home* **(C08P, female patient)**

One GP, when presented with a woman with numbness in her face and arms, diagnosed stress and told the patient that she should go home and have a good cry. This misdiagnosis was confusing for the patient, who felt initial reassurance but also concern that she had not been diagnosed correctly.

*Well I couldn't understand, I just thought I'll go and see my GP, see what he says, it was just for reassurance really, and of course when he said go home and have a good cry, I thought well you know I must be alright, but I knew in the back of my mind I wasn't.* **(A07W, female patient)**

## Discussion

### Main findings

Delays in seeking emergency medical care were partly dependent on patient-related factors, such as the interpretation of signs and symptoms. Fear and denial, reticence to inconvenience medical services, and the desire to contact family members, friends or their own GP initially contributed to delay in reaching hospital quickly. The fact that people often experienced their stroke not as a sudden dramatic event but as a complicated set of disparate symptoms, also contributed. The Act FAST campaign seemed to have raised awareness of stroke in some patients but has not necessarily translated into faster hospital admission in this group, particularly if the patient experienced different symptoms to those highlighted by the campaign. While the presence of a witness was a positive factor in ensuring emergency care services were contacted, there was also an inherent delay if the patient contacted the witness first.

### Strengths and limitations

This study builds on previous work in this area and is strengthened by the inclusion of witnesses in the sample who were able to provide their own accounts of the help-seeking behaviours of patients after stroke. A further strength of the study is that patients and witnesses were purposively sampled to include those who had made immediate contact with the different medical services and those who had delayed.

One of the limitations of this type of study is that the data represent participants' own accounts and perceptions of events. People are arguably more likely to report distressing events than positive, but uneventful, proceedings. Participant accounts must therefore be understood within constraints of interpretation that include differential understandings and perspectives of the qualitative researcher and participant. In recognition of this we have presented sufficient extracts of raw data to accompany the results.

There is potential for recall bias in stroke patients because of the possibility of cognitive impairment. However, the fact that the patients in this study tended to have had less severe strokes, and were able to consent to take part and participate in an interview within 14 days of their stroke event, indicates that impairment was limited amongst this group of patients. The population of stroke patients interviewed did not include those with severe stroke, for obvious reasons, but a number of the witness accounts involved patients with more severe symptoms. It was not possible within the constraints of the study to go back to the participants in order to validate their responses, but multiple coding allowed exploration of potentially competing interpretations of the data.

### Relationship to existing knowledge

Level of stroke severity is an important factor influencing delays for a number of reasons. Firstly, people are more likely to recognise a more severe stroke, as the symptoms are likely to be typical of what many people expect of a stroke (one-sided weakness and speech difficulties) and consistent with the symptoms portrayed in the Act FAST campaign. [17] Secondly, both patients and witnesses are more likely to consider severe stroke to require urgent contact with emergency medical services. The association between stroke severity and early admission to hospital is well recognised and has been found in other studies. [18]–[20] A previous study looking at perceptual, social and behavioural factors has shown that the factors linked with faster hospital admission include perceiving symptoms as severe; a third party noticing the symptoms; and advice by others to seek help. [5] There is thus a need to address the issue of symptom interpretation and perception of stroke severity in interventions to accelerate emergency admission to hospital.

There was reluctance on the part of some patients at the onset of symptoms to accept that they were having a stroke. Patients looked for alternative explanations. Many would ‘wait and see’ if symptoms resolved. This was particularly the case if they did not feel ill, if the symptoms were mild, non-specific, emerged over time or if symptoms did not fit some or all of those described by the Act FAST campaign materials. Most of the patients in this study had no previous personal history of stroke, did not recognise their symptoms as being stroke-related and therefore did not regard them as serious. Moreover, even those who had suffered a previous stroke, and suspected they were having symptoms of new stroke, did not necessarily seek emergency medical assistance. This supports previous work, which has found that, whilst patients with previous stroke were much more likely to be able to identify their symptoms as stroke, this did not mean that medical attention was sought any earlier. [21] Therefore, knowledge of the signs and symptoms may not be sufficient to determine whether emergency medical services are contacted immediately after the onset of symptoms. Interventions should be developed to influence future behaviour among existing stroke patients, who are at increased risk of stroke.

While many patients in this study realised what was happening to them, some of them were unaware of the seriousness or complications of stroke and therefore of the necessity to treat it as a medical emergency. Previous studies have found that knowledge and awareness of stroke symptoms does not necessarily result in an appropriate response or faster admission to hospital. [21]–[27] Most of the patients in this study were older (range from 41 to 86 years, with most participants over 70 years) and it has been found that knowledge of stroke symptoms is lowest in older age groups (i.e. those at greatest risk of stroke). [28]–[30] Furthermore, the presence of co-morbidities in older patients is likely to make symptom recognition more complicated and thus lead to delays.

While the Act FAST campaign appeared to have raised awareness of stroke among study participants, there remained some confusion about what constituted a stroke, especially where the stroke experience varied from the symptoms described on the advertisements/posters. The focus of the campaign on three defined symptoms appeared to have had the unintended consequence of introducing delays in accessing emergency medical service in those not experiencing these classical symptoms/signs. This is in line with previous studies that found that 11% of strokes do not present with FAST symptoms [31] and that faster hospital admission occurs when symptoms present as expected. [32] The Act FAST campaign [11] aims to improve motivation to get patients to hospital quicker by associating speed with improved outcomes (though not with specific treatments). Knowledge acquisition was also described as experiential, and therefore narratives including ‘real’ people (for example in adverts and soap operas) were perceived as a powerful way of conveying information. Market research has shown that public awareness about stroke symptoms and the need to call emergency medical services increased immediately following the Act FAST campaign. [33] However, reliable data about the impact of the campaign on action taken by patients and witnesses at the onset of stroke symptoms is lacking, and evidence from the UK and elsewhere suggests that recognition of symptoms as stroke and knowledge that stroke is a medical emergency does not determine help seeking behaviour. [5], [8], [12], [29], [34]–[40]

How symptoms are acted upon depends on how they are perceived and the way in which they are defined, which are in turn influenced by peoples' prior experiences of illness, as well as the cultural norms and values of the community in which they live. [41] In common with other work, the patients in this study (particularly those with less severe strokes) experienced a range of symptoms and many did not want to accept that they were ill or were reluctant to accept illness as part of their life. [42] The reluctance of patients to contact emergency medical services meant that it was often family members who took the initiative in seeking help. The small number of patients who immediately contacted emergency medical services tended to be those who had witnessed the effects of stroke in close family members and wanted to reduce the effects of stroke as much and as quickly as possible. However, some patients in the study had previously suffered negative experiences in hospital, and their fear of hospitalisation outweighed their desire to seek treatment.

Most of the participants in this study were older people and did not want to ‘make a fuss’ or use resources unwisely. These are views often held by older people. [43] The impact of denial also played a part – to call for professional help was to acknowledge the stroke and thereby its potential consequences. The way in which patients responded to stroke symptoms involved interpreting their symptoms, evaluating possible responses and deciding what to do about them. The patient's response was rarely made without consultation with family members or friends. Interestingly, this even occurred with several patients who had previous strokes and knew what was happening to them. Seeking the support and reassurance of loved ones during a frightening and confusing time is an understandable reaction, but this process of lay referral [35] inevitably results in delays in seeking professional help, particularly if the relative or friend initially has to travel to the patient's home to assess the situation.

In keeping with the desire to seek reassurance from knowledgeable others, a number of participants believed that consulting with their GP was the most appropriate immediate action when they experienced symptoms of stroke. They also believed their GP was a known and trusted source of medical information and advice. However, delays resulted from the time taken to make and attend appointments, the time taken to access ambulance services from the GP's surgery and, in some cases, apparent lack of recognition by GPs of stroke symptoms or failure to treat stroke as an emergency.

Comparisons can be drawn with previous work following the introduction of thrombolysis for myocardial infarction (MI). A study looking at the reasons people delayed calling for help following MI found that 40% of cases had a pre-hospital delay time of more than four hours. Reasons for this included: non-recognition of the symptoms as serious; hoping the symptoms would abate spontaneously; and calling a GP as the first course of action. [44] As delays in time from onset of symptoms to hospital admission and delivery of treatments is crucial in both conditions, it is possible that using strategies to improve awareness of stroke similar to those deployed for myocardial infarction, including recognition of symptoms and the need to treat as an emergency, may result in similar improvement and speedier access to treatment. More targeted interventions for stroke, defined by the appropriate involvement of ‘at risk’ patients in their design, should be developed in order to ensure the translation of symptom knowledge and recognition of the need for an emergency response into appropriate action.

## Conclusions and Implications

Our results provide a picture of the complexities and the multiple factors involved in shaping the decision making process in witnesses and people with stroke symptoms.

The way that patients interpret their symptoms, the presence of a witness and prior knowledge of stroke symptoms and consequences all influenced the decision to make rapid contact with emergency medical services. Perceptions of the roles of primary care and emergency medical services, reticence to burden others and perception of the potential impact of stroke are all factors which have important implications for the design of interventions to increase the proportion of patients with stroke arriving early enough to receive hyper-acute treatments, such as thrombolysis.

**Figure 1:**
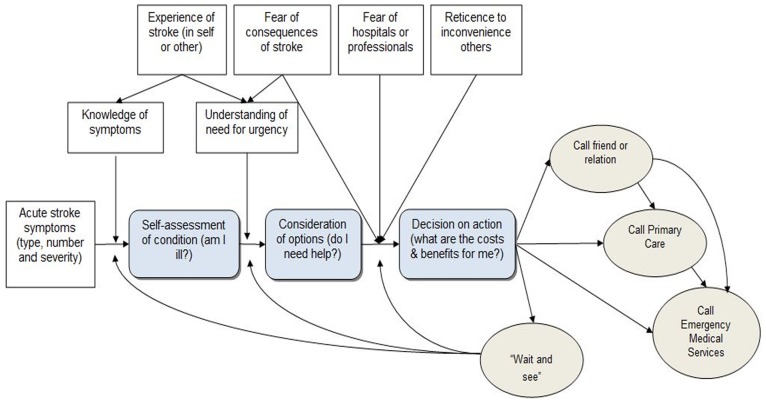
Flow chart showing hypothesised help-seeking decision pathway for patient with acute stroke symptoms.

This complex web of factors, which influences the speed with which a patient with stroke is transferred to emergency medical care and thus the speed with which they can receive thrombolytic therapy, if appropriate, can be summarised diagrammatically (Figure 1). Such an analysis of the causal pathways offers insights into the points at which decisions are made and thus the targets (and specific nature) of potential interventions to change patient and professional behaviour to reduce delays. [20] For example, the patient might not recognise their symptoms as stroke or knows there is something wrong and decides to make an appointment with their GP. However, it is acknowledged that figure 1 may present an incomplete or over simplistic picture. For example, it focuses on the patient, yet the role of a witness may represent a parallel and interacting decision making process. Nevertheless, it offers an aid to determining and prioritising future research on intervention development. Potential interventions may include continued awareness-raising and reinforcement of the messages to legitimise the use of emergency medical services for suspected stroke to inform people not only about the possible meaning of their symptoms, but also the importance of contacting emergency medical services immediately and the reasons for this. Further research is being undertaken to develop interventions to be delivered by primary care staff for those considered ‘at risk’ of stroke. Such interventions should also have the effect of raising awareness amongst such staff about the need for an immediate emergency medical response.

## Supporting Information

Checklist S1 **Consolidated criteria for reporting qualitative studies (COREQ).**
(DOCX)

Protocol S1 **DASH I: Qualitative study of the reasons why people do, or do not, contact emergency medical services following the onset of stroke symptoms.**
(DOCX)
