# Supplementary material for: Why People Do, or Do Not, Immediately Contact Emergency Medical Services following the Onset of Acute Stroke: Qualitative Interview Study
Source: PLoS One. 2012 Oct 4;7(10):e46124. doi: 10.1371/journal.pone.0046124 (PMC3464281; doi:10.1371/journal.pone.0046124)
Supplement: Protocol S1 — DASH I: Qualitative study of the reasons why people do, or do not, contact emergency medical services following the onset of stroke symptoms. (DOCX) [file pone.0046124.s002.docx]

# DASH I: Qualitative study of the reasons why people do, or do not, contact emergency medical services following the onset of stroke symptoms

Joan E Mackintosh^1^, Madeleine J Murtagh^2^, Helen Rodgers^3^, Richard G Thomson^1^, Gary A Ford^3^, Martin White^1 4^

1. Institute of Health & Society, Faculty of Medical Sciences, Newcastle University, UK
2. Department of Health Sciences, College of Medicine, Biological Sciences and Psychology, Leicester University, UK
3. Institute for Ageing & Health, Faculty of Medical Sciences, Newcastle University, UK
4. Fuse, UKCRC Centre for Translational Research in Public Health, Newcastle University, UK

## 1. Background and Purpose

Thrombolysis with intravenous tissue Plasminogen Activator (tPA) when given to carefully selected patients within 4.5 hours of the onset of symptoms of acute ischaemic stroke reduces the risk of dependency.^2,5^ It is estimated that at least 10% of stroke patients may be eligible for thrombolysis and NICE has a target implementation that within 5 years 10% of ischaemic stroke patients < 80 yrs should be treated with tPA.^5^ Currently less than 0.7% of patients with acute ischaemic stroke in the UK receive this treatment (although for individual centres in the UK 5-10% of acute ischaemic stroke patients admitted to their service receive this treatment).^4,8^ There are two factors that contribute to this poor access to acute stroke treatment: an inadequate emergency care response; and insufficient public awareness of the signs and symptoms of stroke or the need to treat it as a medical emergency.

Low thrombolysis rates are in part due to delays in seeking medical attention following the onset of symptoms. In 2002 the median time between onset of symptoms and arrival to hospital in the UK was 6 hours with only 37% arriving within 3 hours.^1^ Early recognition and rapid response to the symptoms of stroke by patients and bystanders are important dimensions of improving outcome following stroke.^3^

In a recent systematic review^6^ we found that, while patients and the general public in the UK regarded stroke as a serious condition which required emergency treatment, they had limited knowledge of the symptoms of stroke. In addition, although members of the general public reported that they would call an ambulance in the event of a stroke, in practice both patients and witnesses often initially contacted a general practitioner, which had a major impact upon time from symptoms to admission. A significant number of patients and witnesses waited to see if symptoms resolved. Thus although people report they consider stroke to be an emergency condition, observed behaviour suggests this knowledge does not result in an appropriate response.

A key component of the English National Stroke Strategy is to ensure that patients with acute stroke are treated as a medical emergency.^3^ The strategy seeks to ensure that ‘members of the public and health and care staff are able to recognise and identify the main symptoms of stroke and know it needs to be treated as a medical emergency’. Over the next 3 years £12 million will be invested in a multi-faceted strategy to improve awareness of, and response to, stroke.

One of the objectives of the broader NIHR-funded DASH (Developing and Assessing Services for Hyperacute stroke) programme of research being undertaken by our research team, is to provide evidence to support this work by seeking to understand why patients and those who witness a stroke in the UK do or do not seek emergency help following a stroke. As part of that programme, this study will investigate how and why emergency medical services are accessed at the onset of (acute) stroke by people with stroke and ‘witnesses’ to the stroke e.g. family members/friends/carers/bystanders who were present at the time of stroke or who found the patient with stroke and made initial contact with medical services. We define ‘access to emergency services’ as deciding whether, or not, to immediately call emergency medical services. The results of this work will inform the development of interventions to increase awareness of stroke symptoms and the accessing of stroke services at the onset of stroke among stroke patients and the general public.

## 2. Aim of DASH 1 Qualitative Study

To understand the reasons individuals contact or delay contacting emergency medical services in response to symptoms of stroke in order to support the development of an intervention for raising patient and public awareness of stroke symptoms; thus increasing rapid access to hyper-acute stroke services for people with acute stroke.

## 3. Aim of this work

To identify, describe and understand how and why patients and witnesses did, or did not, seek immediate emergency medical assistance following the onset of stroke symptoms.

## 4. Timescale

The qualitative work with stroke patients and witness(es) will take 16 months from the 1^st^ November 2008. This consists of two months to obtain ethical and R&D approvals, six months to recruit and interview patients and witnesses, and eight months for analysis and writing up.

## 5. Study Methods

We will conduct a qualitative study of reported actions in accessing, or not accessing, emergency medical services by stroke patients and ‘witness(es)’ at the onset of stroke symptoms. This will be undertaken through the use of semi-structured interviews of stroke patients and witness(es).

The topic guides for the interviews will be developed in collaboration with people with stroke, identified through the North East Stroke Research Network.

A situational analysis^7^ will be used in the analysis of interview transcripts. Situational analysis builds on traditional grounded theory and involves constructing the situation of inquiry empirically through mapping. Situational maps lay out the major human, non-human, discursive and material elements in the research situation of concern and provoke analysis of relations among them, thus allowing the deeper exploration of themes which are raised by participants or are implicit in the interview transcripts.

## 6. Study Design Overview

We will undertake face-to-face interviews with:

- Patients within two weeks of stroke;
- ‘witness(es)’ who were instrumental at the time of the stroke in accessing, or not accessing, emergency services.

## 7. Setting

Stroke units in NHS North of Tyne; Newcastle upon Tyne Hospitals NHS Foundation Trust and Northumbria Healthcare NHS Foundation Trust.

## 8. Sample

Up to 48 participants (24 patients and 24 ‘witnesses’) who made the initial call to NHS services which led to the patient being admitted to hospital following a stroke event. The sample will comprise the individuals who had a stroke, and ‘witnesses’ (those who were present at the time of stroke/found the patient and made the initial telephone call), giving a total of 48 separate events.

The sample of 48 will consist of 4 categories of participants (the caller, either patient or witness, for an individual with a stroke event) who:

- was admitted to hospital **via emergency care** (999 or direct to A&E) **within one hour** of the onset of stroke
- was admitted to hospital **via emergency care** (999 or direct to A&E) **after one hour** following the onset of stroke.
- was admitted to hospital **via GP or other routes within one hour** following the onset of stroke ,
- was admitted to hospital **via GP or other routes after one hour** following the onset of stroke .

Those who had a stroke during their sleep where the time of onset of symptoms is not known **will not be excluded**, but will be included to ascertain what action they took on becoming aware of the stroke event, i.e. upon wakening.

As patients who are too ill will be excluded from participating, it is likely that our patient sample who made the call themselves will include more patients with less severe stroke events, However, it is less likely that patients with severe stroke will have made calls and more likely that they will have been made on their behalf by informants who will be our witness sample. Thus the overall sample will include both severe and less severe events.

**Participant sample matrix**

|  | Service accessed via 999 or direct to A&E | Service accessed by other route (e.g. GP & NHSDirect) |
| --- | --- | --- |
| Patients admitted within 1 hour of stroke onset | 12 | 12 |
| Patients admitted after 1 hour of stroke onset | 12 | 12 |

As this is a qualitative study the sample size is not designed to provide statistically representative samples, but will be large enough to capture the range of experience and actions for each section of the sample matrix. If this is felt not to have occurred we will sample until there is no new data.

## 9. Identification and Recruitment

Research collaborators in each of the three stroke units will assist in recruitment and assessment of those eligible to participate in the study. The stroke units in this study are involved in other aspects of the broader DASH programme of research.

Up to 48 patients and witnesses, representing 48 different events, will be recruited across three sites.

*Patient recruitment*

Potential patient participants will be identified by clinical staff on admission to the stroke unit and approached by Stroke Research Network (SRN) research nurses on the stroke unit 1-2 days post-stroke. The SRN research nurse, who will have been briefed on the research study, will ask patients if they were the person who made the call leading to hospital admission and if so whether they are willing to participate in the study. SRN research nurses will provide patients with information (written and verbal) to allow the patient to make an informed decision about participation. The research team will be notified when a patient has agreed to take part in the study and a meeting will be arranged with the researcher to provide further information and/or an interview (as per the patient’s wishes). Interviews will take place on the stroke unit for in-patients. Those who have been discharged early will be given the choice of being interviewed either in their own home, on the stroke unit or at the university. Prior to the interview commencing, written consent will be sought from the patient. (see Appendix C). Patients will be interviewed at 7 +/- 2 days post-stroke.

Patients will only be interviewed if they are deemed to have capacity adequate for engagement in decision-making at the time of enrolment and if they also have capacity for interview at the time of consent. Capacity will be determined by a member of the clinical team who is responsible for the care of the patient. All patients with diagnosis of acute stroke will be eligible, irrespective of treatment received.

*Witness Recruitment*

When a patient is approached by the SRN research nurse and asked if they were the person who made the call leading to hospital access, if they say that a witness made the call, then that witness will be approached to ascertain if they would be willing to be contacted about participation in the study. They will be given a copy of the witness information sheet (appendix B). If they indicate that they are willing to participate, the research associate will make contact and provide any further information required/answer any questions and arrange a time and place for the interview. We will also recruit witnesses by direct contact through SRN research nurses with accompanying carers/relatives to ensure we collect data from people involved in making contact with NHS services after severe stroke events. We will follow the procedures for recruitment as for patients, with a separate written consent form for witnesses (Appendix D). Witnesses will be given the choice of being interviewed in their own home, at the hospital or at Newcastle University.

Patients and witnesses will be interviewed independently. It is anticipated that a patient may request that a carer/significant other person is present with them during the interview, but as the categories ‘patient’ and ‘witness’ are mutually exclusive this should not impact negatively on the study

## 10. Exclusion Criteria

Patients will be excluded if they are considered by their consultant to lack the mental capacity to be able to provide informed consent to participate in the study. Witnesses are not anticipated to be excluded except with reference to the sampling matrix. However, both patients and witnesses under the age of 18 and those not considered capable of giving informed consent, either by their consultant or the researcher at the time of the interview, will be excluded. Those individuals who have severe communication difficulties will also be excluded so as to avoid causing them distress. We will exclude those not able to communicate verbally in English.

## 11. Data Collection

*Detailed steps:*

Potential interviewees will be identified at admission or during clinical contact within 1-2 days post-stroke by SRN research nurses. The responsible clinician will assess patient capacity for involvement in the research according to the Mental Capacity Act 2005.

If patients are deemed to have capacity, SRN research nurses will ask them if they were the person who made the call leading to hospital access. If so the SRN nurses will tell the patient about the study and provide potential participants with a copy of the patient information sheet (appendix A). If the patient was not the person who made the call leading to hospital access they will be asked who did. If the patient is not capable of providing this information the SRN research nurse will ask those family and friends present to identify the individual and will provide potential participants with a copy of the witness information sheet (appendix B).

After a period for reflection (24-48 hours), the SRN research nurse will approach potential patient participants to ascertain willingness for involvement. The SRN research nurse will alert the DASH research team in order that they may visit the ward and be introduced to the potential participant. Potential participants may also contact researchers directly as per information sheet details if desired. The Research Associate will be introduced to patients by a clinical member of staff and make first face to face contact with potential interviewees during days 3-5. At this point the research associate will confirm the potential interviewee has received and understood the information sheet detailing the study, answer any questions and seek their willingness to proceed. If willing, an interview time and place will be arranged. If not obtained at first contact, written consent will be sought at time of interview at 7 +/-2 days post-stroke. The patient may choose to have another person present, therefore interviews may take place with more than one person. ‘Witness’ details will be elicited from the patient (or their families/friends) and they will be given the information sheet by the SRN research nurse. If they assent to be contacted, the research associate will make contact, confirm they have received and understood the information sheet detailing the study, answer any questions and seek their willingness to proceed. Interview and consent procedures will be the same as that for patient participants. Witnesses will be interviewed separately.

Interviews are expected to last between 20 minutes to one hour.

Topic guides will be based on existing evidence and will be developed iteratively in collaboration with patients, stroke researchers and clinicians. Patient and carer representatives from the local Stroke Research Network and DASH groups will be asked to review the topic guide.

Interviews will include questions about:

- their views about what was happening and what they should do about it;
- their actions at the onset of stroke symptoms and why;
- what their concerns and fears were at that time;
- their knowledge of the signs and symptoms of stroke at that time;
- awareness of stroke as a medical emergency at that time;
- their knowledge about available treatment for stroke;
- what advice on signs and symptoms they sought at that time;
- what knowledge of medical services for stroke they had at that time;
- their attitudes towards medical services for stroke;
- their views about the benefits or disadvantages of going to hospital;
- barriers to accessing stroke services.

The interview will be semi-structured and the interview structure will be flexible to take account of the different needs and perspectives of participants.

The topic guide will be developed iteratively throughout the interviews, which will continue until the sample matrix requirements are reached. All of the interviews will be digitally recorded (with the respondents’ consent) and fully transcribed. The transcripts will provide the formal data for analysis. Transcripts will be interpreted iteratively, developing themes (or categories) within respondents’ discourse, using NVivo data analysis software.

## 12. Analysis

All interviews will be audio-recorded (with respondents’ consent) and transcribed. Transcripts of the interviews plus detailed post-hoc field notes from observations taken during the research will form the data for analysis. Transcripts will be checked for accuracy against the recordings and then anonymised. Analysis of interview transcripts will be undertaken utilising situational analysis^7^

## 13. Validity

As a qualitative study, this research sets out to understand the range of views held by people with stroke and witnesses. Purposive sampling will allow theoretical generalisations to be made about how and why stroke patients and witnesses access emergency care for acute stroke. Validity will be achieved through saturation of themes, exposition of methods, attention to negative cases, member checking, triangulation and reflexivity.

## 14. Ethics and consent

Ethical approval for the study will be sought from the Local NHS Research Ethics Committee. The approval/support of the relevant Hospital Trusts, along with those of the appropriate local authorities, will be sought prior to the study, as will that of the individual hospital departments. Newcastle upon Tyne Hospitals NHS Foundation Trust will act as study sponsor. The study will comply with the Research Governance Framework for Health and Social Care.

Separate information sheets will be provided for patients and witnesses (Appendices A and B). If requested, a copy of the research protocol will be supplied. Written consent to audio-record and participate in the interviews will be sought from patients and witnesses at the time of the interview by a member of the research team (Appendices C and D). The interviewer may halt the interview at any stage if it is felt that the participant is no longer comfortable or does not wish to proceed any further.

All data will be kept securely in password protected computer systems or locked filing cabinets, and anonymised at the earliest opportunity. There will be full compliance with the Data Protection Act to ensure participant confidentiality. Written, informed consent will be requested from study participants to participate.

### 15. Safety

The University has developed safety protocols to guide staff undertaking fieldwork, especially in participants’/clients’ own homes. These will be adhered to strictly throughout the study.

**References**

1. Harraf F, Sharma AK, Brown MM, Lees KR, Vass, RI and Kalra L. A multicentre observational study of presentation and early assessment of acute stroke. BMJ 2002;325; 1-5

2. Hacke W, Kaste M, Bluhmki E, Brozman M, Dávalos A, Guidetti D, Larrue V, Lees KR, Medeghri Z, Machnig T, Schneider D, von Kummer R, Wahlgren N, Danilo Toni D, for the ECASS Investigators. Thrombolysis with Alteplase 3 to 4.5 Hours after Acute Ischemic Stroke. NEJM, 2008; 359:1317-1329

3. Department of Health. *2007 National Stroke Strategy*, Department of Health Publications, London

4. Royal College of Physicians. National Sentinel Stroke Audit Report 2004. Prepared on behalf of the Intercollegiate Stroke Working Party. London, RCP, 2005

5. National Institute for Health and Clinical Excellence. *NICE clinical guideline 68. Stroke: diagnosis and initial management of acute stroke and transient ischaemic attack (TIA)*, National Institute for Health and Clinical Excellence, London, July 2008

6. Lecouturier J, Murtagh M, Thomson R, Ford G, White M, Eccles M, Rodgers H, Response to symptoms of stroke and TIA in the UK: A systematic review.

7. Clarke, A. (2005). *Situational analysis.* Thousand Oaks, CA: Sage.

8. Rodgers H, Thomson R. Functional status and long term outcome of stroke. BMJ. 2008; 336: 337-342.
